# Supplementary material for: Multidimensional third-generation sequencing of modified DNA bases allows interrogation of complex biological systems
Source: Nat Commun. 2025 Jul 1;16:5676. doi: 10.1038/s41467-025-60896-x (PMC12215381; doi:10.1038/s41467-025-60896-x)
Supplement: Supplementary file 1 — Supplementary Information [file 41467_2025_60896_MOESM1_ESM.pdf]

Supplementary Information

Multidimensional third-generation sequencing of modified DNA bases allows interrogation of complex biological systems.

Serena S. David[1]†, Brendan A. Pacheco[1]†, Kensei Kishimoto[1], Sam Vantine[1], Kai Hu[1], Haibo Liu[1], Diana L. Davis[1], Hoang Tran[1], Benjamin F. Sallis[2,3], Levi Ali[1], Cole M. Haynes[1], Beth A. McCormick[2,3], Lihua Julie Zhu[1], William A. Flavahan*[1]

[1]Department of Molecular, Cell and Cancer Biology, University of Massachusetts Chan Medical School; Worcester, Massachusetts 01605, USA.

[2]Department of Microbiology, University of Massachusetts Chan Medical School; Worcester, Massachusetts 01605, USA.

[3]Program in Microbiome Dynamics, University of Massachusetts Chan Medical School; Worcester, Massachusetts 01605, USA.

†These authors contributed equally to this work: Serena S. David, Brendan A. Pacheco

*Corresponding author. Email: william.flavahan@umassmed.edu

Contents:

Supplementary Figure 1. Additional details of BSPS oligonucleotide synthesis.

Supplementary Figure 2. Nanopore current characteristics of the thymine analogs, deoxy-Uracil and BrdU.

Supplementary Figure 3. BSPS allows the synthesis of training oligos for mixed heteropolymers of modified cytosines and for abasic sites.

Supplementary Figure 4. Statistical analysis of the predictive power of modified base detection at the single-base and single-read level.

Supplementary Figure 5. Analysis of known standard BSPS oligos with DNAscent.

Supplementary Figure 6. Recurrent Neural Network (RNN) classification of BrdU-containing sequencing reads and comparison to other methods.

Supplementary Figure 7. Statistical analysis of single-base detection of additional modified bases.

Supplementary Figure 8. dU incorporation results in predictable sequencing errors in the standard basecaller.

Supplementary Figure 9. Assembled *Shigella* genomes created without using ^{6m}A detection information.

Supplementary Figure 10. Potential expansions to the BSPS approach.

Supplementary Table 1. Cost estimates for a single BSPS synthesis reaction.

Supplementary Table 2. Details for generated BSPS sequencing libraries.

Supplementary Note 1: Example BSPS protocol.

Supplementary Note 2: Example BSPS data processing commands.

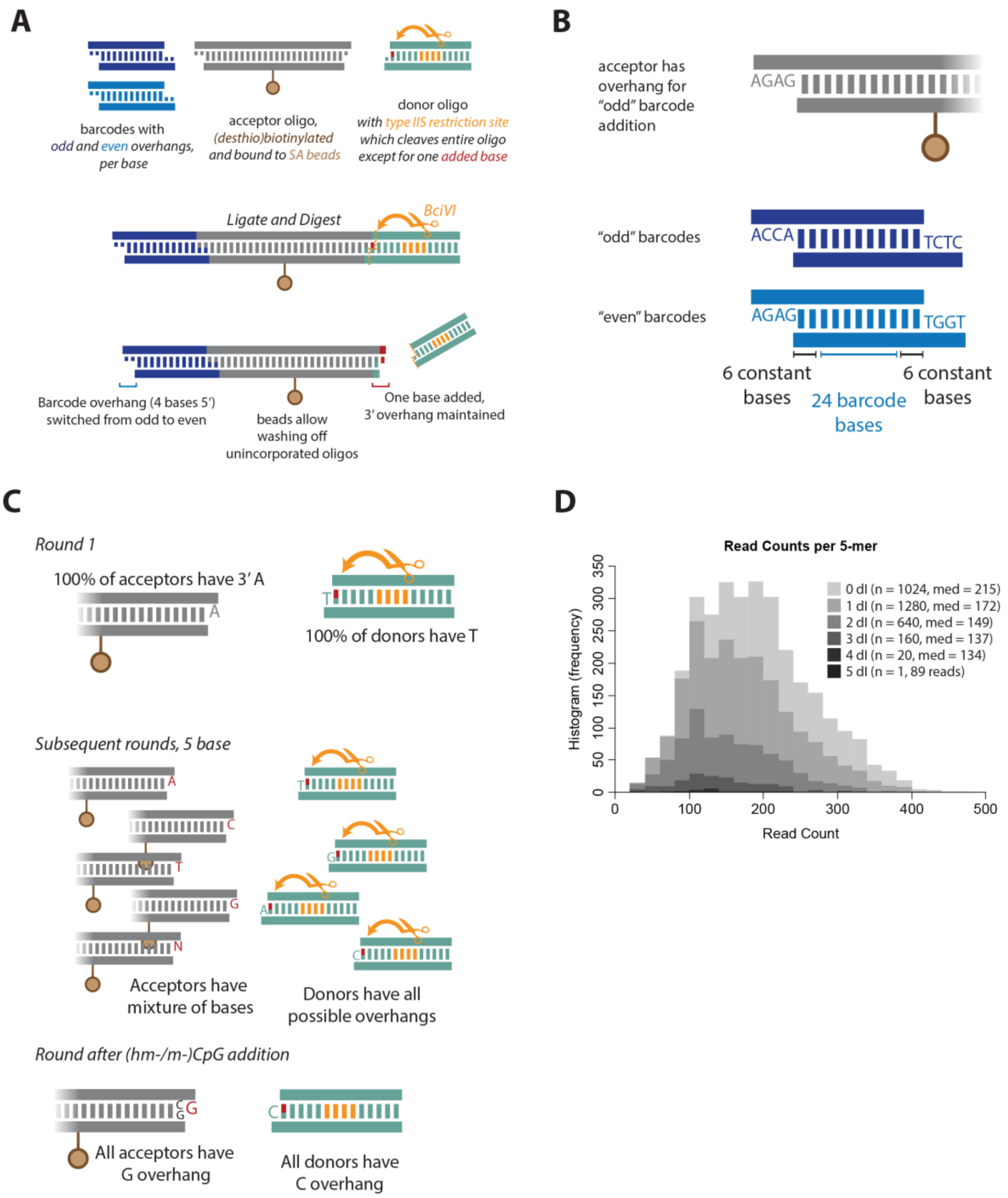

Supplementary Figure 1. Additional details of BSPS oligonucleotide synthesis. **(A)** Schematic depicts ligation reaction performed in each reaction tube (see Fig. 1a). A barcode, with either even or odd overhangs, is ligated to one end of the universal acceptor oligo, while a donor oligo is ligated to the other. The donor oligo contains a type IIS restriction site (for the BciVI enzyme), which will cleave outside its asymmetric recognition site and remove the entire donor oligo except for a single added base. (**B**) Schematic depicts "even round" and "odd round" barcode strategy. The universal acceptor has the initial overhang for an 'odd' barcode, which when ligated will change the overhang of the full synthesized oligo to 'even' and prevent addition of multiple barcodes per round. The barcode structure is also indicated; each barcode contains 6 constant bases on each end, to identify barcode junctions easier, and 24 barcode-unique bases internally. (**C**) Schematic depicts donor oligo pool overhang strategy. In the first round, every acceptor has a single 3' A overhang, so the donor pool has 100% 3' T overhangs. In subsequent rounds, the donor oligo pool contains a mixture of 3' base overhangs corresponding to the overhangs likely present on the growing BSPS oligos. In a five-split synthesis with each base present, each base will be represented in the donor oligos thus allowing for donor addition to each growing BSPS oligo. Following a round where modified CpGs were added to all oligos, the donor oligo pool will have exclusively C overhangs to increase donor addition efficiency. (**D**) Histogram depicts read coverage for all

possible dI-library five-mers (see Figure 2), with the dI-content of five-mers indicated; coverage drops only slightly with increasing dI incorporation.

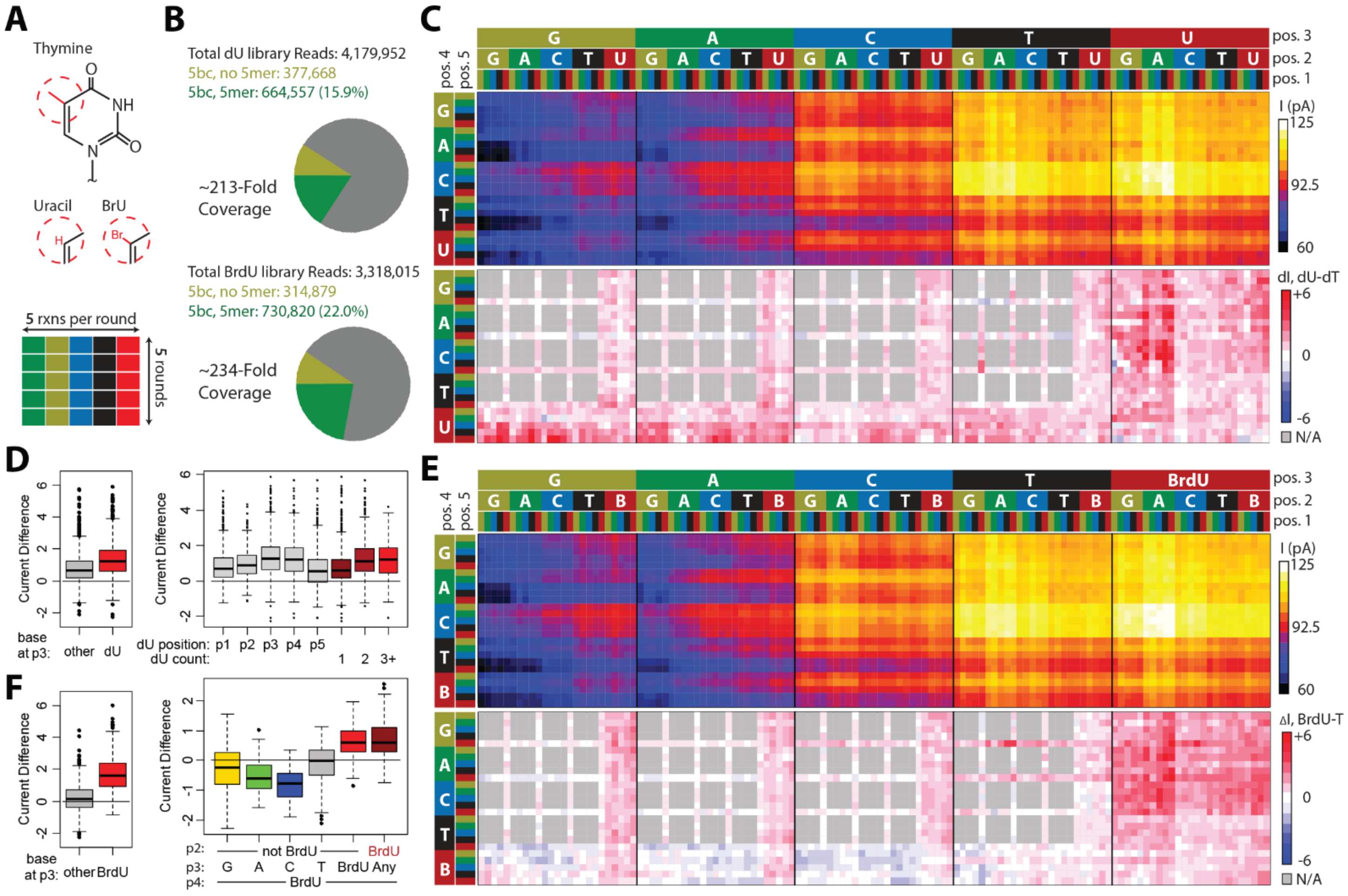

Supplementary Figure 2. Nanopore current characteristics of the thymine analogs, deoxy-Uracil and BrdU. (**A**) The chemical structures of uracil and bromo-uracil are depicted. (**B**) A five-round, five-split synthesis was performed for both dU and BrdU. Characteristics of the synthesized and sequenced libraries are presented. (**C**) Heatmap depicts full current values for all dU-containing five-mers. Top heatmap depicts absolute current value in increasing heat. Bottom depicts current difference of dU-containing fivemers to a reference five-mer with dT in place of each dU. Red depicts a current increase of dU compared to dT, blue depicts a current decrease, and gray indicates five-mers with no dUs. (**D**) Box plots depict current differences of dU-containing five-mers with specific characteristics to dT-containing reference five-mers. Left, five-mers with dU at position 3 are compared to five-mers with dU but not at position 3. Right, five-mers containing dU at the indicated position or the indicated number of dUs are depicted. (**E**) Heatmap depicts the full current values for all BrdU-containing five-mers, as in (C). (**F**) Box plots depict current values of BrdU-containing five-mers with specific characteristics compared to dT reference five-mers. Left, five-mers with BrdU in position 3 are compared to five-mers with BrdU but not at position 3. Right, five-mers with BrdU in position 4 are compared to five-mers with specific bases at position 3, and five-mers that also contain BrdU at position 2. For box plots, center lines depict medians, box limits depict quartiles, whiskers depict 1.5x interquartile range, and points are outliers. Source data are provided as a source data file and supplementary data.

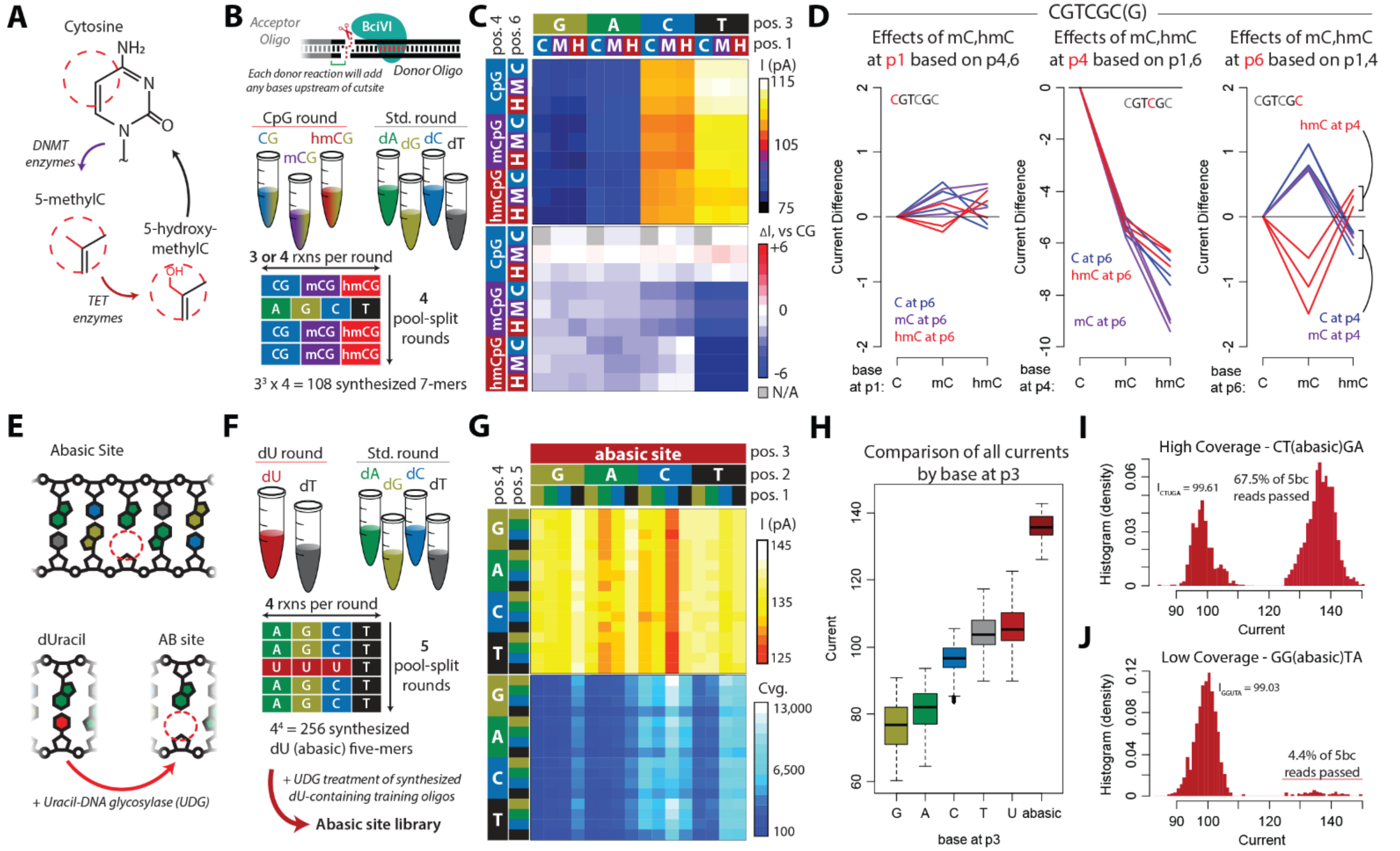

Supplementary Figure 3. BSPS allows the synthesis of training oligos for mixed heteropolymers of modified cytosines and for abasic sites. (**A**) The chemical structures of cytosine, 5-methyl-cytosine, and 5-hydroxymethyl-cytosine are depicted. (**B**) Because of the dynamics of the type IIS restriction enzyme (see Fig. 1B), multiple bases can be added from a single donor oligo. Donor oligos were designed to add either CG, 5mCG, or 5hmCG, and a four round synthesis with three or four splits per round, as indicated by the diagram, was performed to generate 108 synthesized 7-mers. (**C**) Heatmap depicts current values for the indicated 6-mers. Top heatmap depicts absolute current value in increasing heat. Bottom heatmap depicts relative current difference to completely unmodified cytosine reference. (**D**) Traces depict effects of increasing cytosine modification (unmodified > 5-methylation > 5-hydroxymethylation) at the indicated position, based on the surrounding modifications. (**E**) Schematic depicts an abasic (apyrmidinic) site; these can be generated from dU-containing DNA using the enzyme UDG. (**F**) A five-round, four-split reaction was performed to generate 256 five-mers with a dU in position 3 (or the control five-mers with dT), which were treated with UDG to generate an abasic site training library. (**G**) Heatmap depicts current values for five-mers containing an abasic site in position 3. Top heatmap depicts absolute current values, with a different color scale than other heatmaps in previous figures. Bottom depicts read coverage of each five-mer. (**H**) Box plot depicts the current values of five-mers with the indicated base at position 3; every five-mers that has an abasic site at p3 has a higher current value than every five-mer that does not. (**I**,**J**) Histogram depicts observed current values of all five-barcode reads with the indicated five-mer (i.e. including reads that fail the current value check). For box plots, center lines depict medians, box limits depict quartiles, whiskers depict 1.5x interquartile range, and points are outliers. Source data are provided as a source data file and supplementary data.

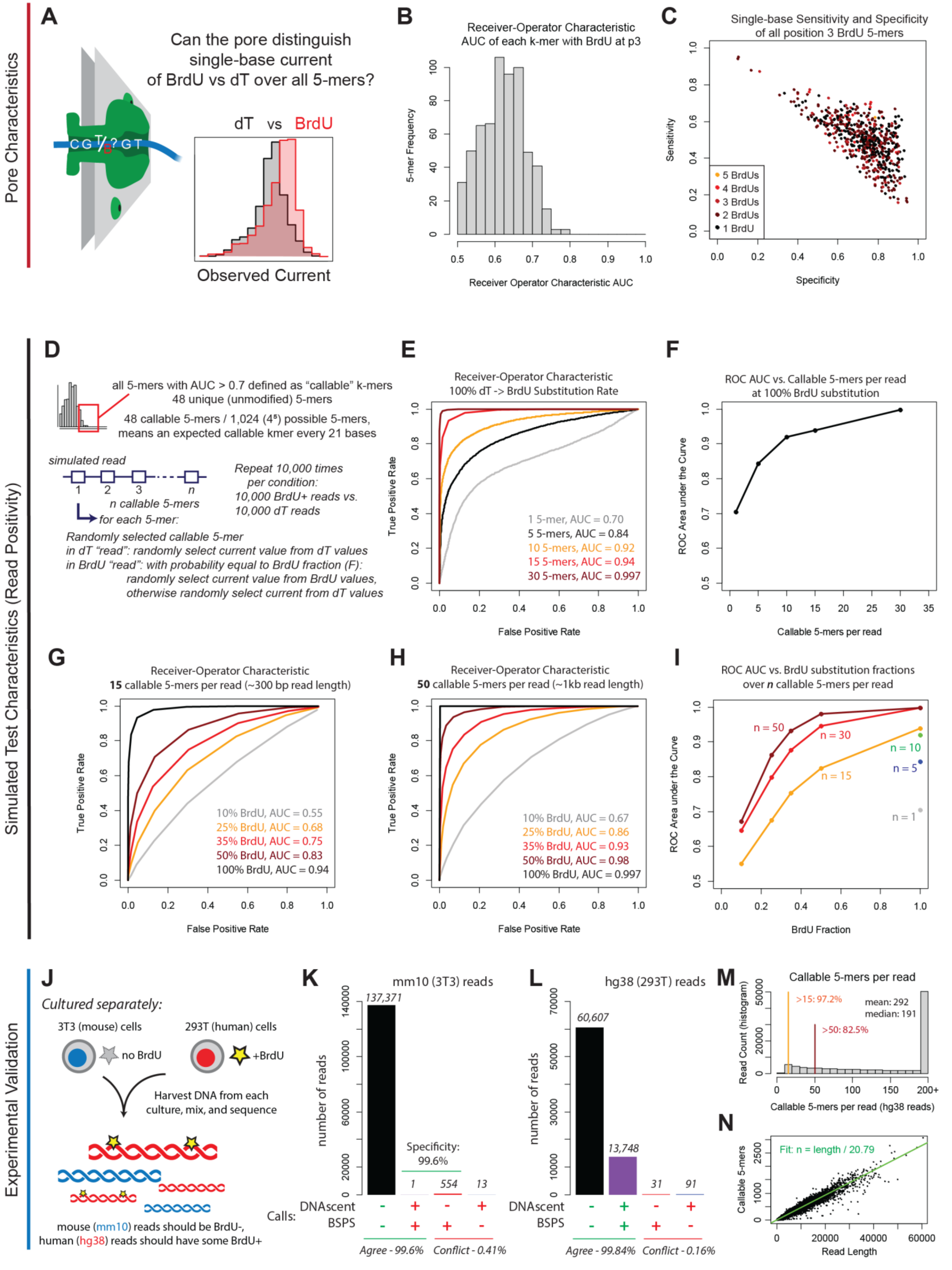
Pore Characteristics
A
Can the pore distinguish single-base current of BrdU vs dT over all 5-mers?
CGT/B?GT
dT vs BrdU
Observed Current
B
Receiver-Operator Characteristic AUC of each k-mer with BrdU at p3
5-mer Frequency
Receiver Operator Characteristic AUC
C
Single-base Sensitivity and Specificity of all position 3 BrdU 5-mers
Sensitivity
Specificity
5 BrdUs
4 BrdUs
3 BrdUs
2 BrdUs
1 BrdU
Simulated Test Characteristics (Read Positivity)
D
all 5-mers with AUC > 0.7 defined as "callable" k-mers
48 unique (unmodified) 5-mers
48 callable 5-mers / 1,024 (4⁵) possible 5-mers, means an expected callable kmer every 21 bases
simulated read
n callable 5-mers
for each 5-mer:
Repeat 10,000 times per condition: 10,000 BrdU+ reads vs. 10,000 dT reads
Randomly selected callable 5-mer
in dT "read": randomly select current value from dT values
in BrdU "read": with probability equal to BrdU fraction (F): randomly select current value from BrdU values, otherwise randomly select current from dT values
E
Receiver-Operator Characteristic
100% dT -> BrdU Substitution Rate
True Positive Rate
False Positive Rate
1 5-mer, AUC = 0.70
5 5-mers, AUC = 0.84
10 5-mers, AUC = 0.92
15 5-mers, AUC = 0.94
30 5-mers, AUC = 0.997
F
ROC AUC vs. Callable 5-mers per read at 100% BrdU substitution
ROC Area under the Curve
Callable 5-mers per read
G
Receiver-Operator Characteristic
15 callable 5-mers per read (~300 bp read length)
True Positive Rate
False Positive Rate
10% BrdU, AUC = 0.55
25% BrdU, AUC = 0.68
35% BrdU, AUC = 0.75
50% BrdU, AUC = 0.83
100% BrdU, AUC = 0.94
H
Receiver-Operator Characteristic
50 callable 5-mers per read (~1kb read length)
True Positive Rate
False Positive Rate
10% BrdU, AUC = 0.67
25% BrdU, AUC = 0.86
35% BrdU, AUC = 0.93
50% BrdU, AUC = 0.98
100% BrdU, AUC = 0.997
I
ROC AUC vs. BrdU substitution fractions over n callable 5-mers per read
ROC Area under the Curve
BrdU Fraction
n = 50
n = 30
n = 10
n = 5
n = 15
n = 1
Experimental Validation
J
Cultured separately:
3T3 (mouse) cells
293T (human) cells
no BrdU
+BrdU
Harvest DNA from each culture, mix, and sequence
mouse (mm10) reads should be BrdU-, human (hg38) reads should have some BrdU+
K
mm10 (3T3) reads
number of reads
137,371
Specificity: 99.6%
1
554
13
Calls: DNAscent
BSPS
Agree - 99.6%
Conflict - 0.41%
L
hg38 (293T) reads
number of reads
60,607
13,748
31
91
DNAscent
BSPS
Agree - 99.84%
Conflict - 0.16%
M
Callable 5-mers per read
Read Count (histogram)
>15: 97.2%
>50: 82.5%
mean: 292
median: 191
Callable 5-mers per read (hg38 reads)
N
Fit: n = length / 20.79
Callable 5-mers
Read Length

Supplementary Figure 4. Statistical analysis of the predictive power of modified base detection at the single-base and single-read level. (**A**) Generation of BSPS libraries permits statistical characterization of the modified base discrimination potential of the nanopore itself. (**B**) Histogram depicts the Area Under the Curve (AUC) of the Receiver-Operator Characteristic (ROC) for the single-base detection of BrdU in each 5-mer with a BrdU in position 3. (**C**) Scatterplot depicts the best sensitivity and specificity for each 5-mer with a BrdU in position 3, as in (B). (**D**) Schematic depicts setup of statistical analysis of simulated reads. Simulated reads were assembled with randomly chosen datapoints from sequencing of the BSPS library, as described, and analyzed for sensitivity and specificity. (**E**) Plot depicts the Receiver-Operator Characteristics (ROC) for simulated reads of a given number of *k*-mers, at 100% dT to BrdU substitution rate. AUCs are indicated in legend. (**F**) Plot depicts the ROC AUC of simulated reads of the indicated length at 100% BrdU substitution, as shown in (E). (**G**) Plot depicts the ROCs for simulated reads with 15 callable 5-mers (corresponding to roughly 300bp read length/region of analysis), at the indicated dT to BrdU substitution rates. AUCs are indicated in legend. (**H**) As (G), but for simulated reads with 50 callable 5-mers (corresponding to roughly 1kb read length/region of analysis). (**I**) Plot depicts the ROC AUC of simulated reads of simulated reads of the indicated length at various levels of BrdU substitution. (**J**) Schematic depicts setup of experimental validation. Mouse cells (3T3s) were grown without BrdU, and human cells (293Ts) were cultured separately in the presence of 100 μM BrdU. DNA was harvested from each cell culture, combined, and sequenced, before being analyzed for BrdU presence by either DNAscent version 3.1.2 or z-score derived from BSPS values. (**K**) Analysis of mm10-aligning mouse reads shows a high degree of concordance between DNAscent and BSPS-based analysis. (**L**) Analysis of hg38-aligning human reads shows a high degree of concordance between DNAscent and BSPS-based analysis. (**M**) Histogram depicts the number of callable 5-mers (see (D)) present in each read from the experiment; the vast majority of reads have significantly higher numbers of callable 5-mers than required for accurate BrdU detection. (**N**) Plot depicts the relationship between the number of callable 5-mers per read and read length; the calculated assumption of ~21 bases per callable 5-mer seems accurate.

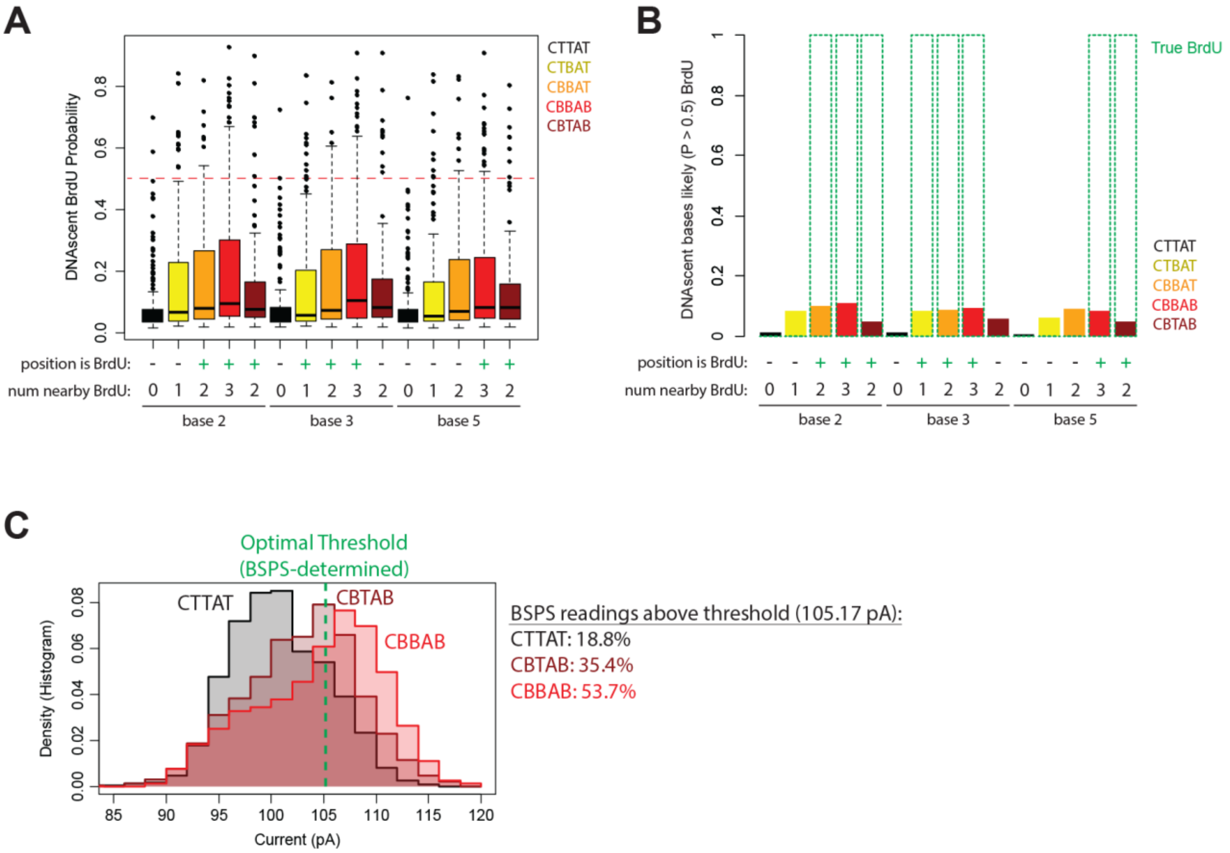

Supplementary Figure 5. Analysis of known standard BSPS oligos with DNAscent version 3.1.2. (**A**) BSPS reads of the indicated encoded 5-mer were analyzed with DNAscent version 3.1.2, and the BrdU probability of the bases at the indicated position is plotted. Whether that specific base is BrdU in the 5-mer and the number of BrdUs in the 5-mer is indicated. Notably, BSPS reads do not contain BrdU anywhere except the encoded 5-mer; the number of nearby BrdUs is also the total number of BrdUs in the read. (**B**) Plot depicts the fraction of bases called as likely BrdU (probability > 50%) by DNAscent, as per (A). Green dashed lines indicate the true BrdU fraction of bases at the indicated read. Overall, these analyses demonstrate that single-base detection of a dT/BrdU substitution is challenging, but read- or region-level analysis performed by analyzing multiple 5-mers together (see Ext. Data Figure 4) allows for accurate detection of reads or read regions with BrdU incorporation. (**C**) Histograms depict current distributions for selected 5-mer sequences, as characterized by BSPS libraries. Indicated are the percentage of reads above the optimally calculated threshold by ROC AUC (see Supplementary Figure 4) for each 5-mer. For box plots, center lines depict medians, box limits depict quartiles, whiskers depict 1.5x interquartile range, and points are outliers. Source data are provided as a source data file.

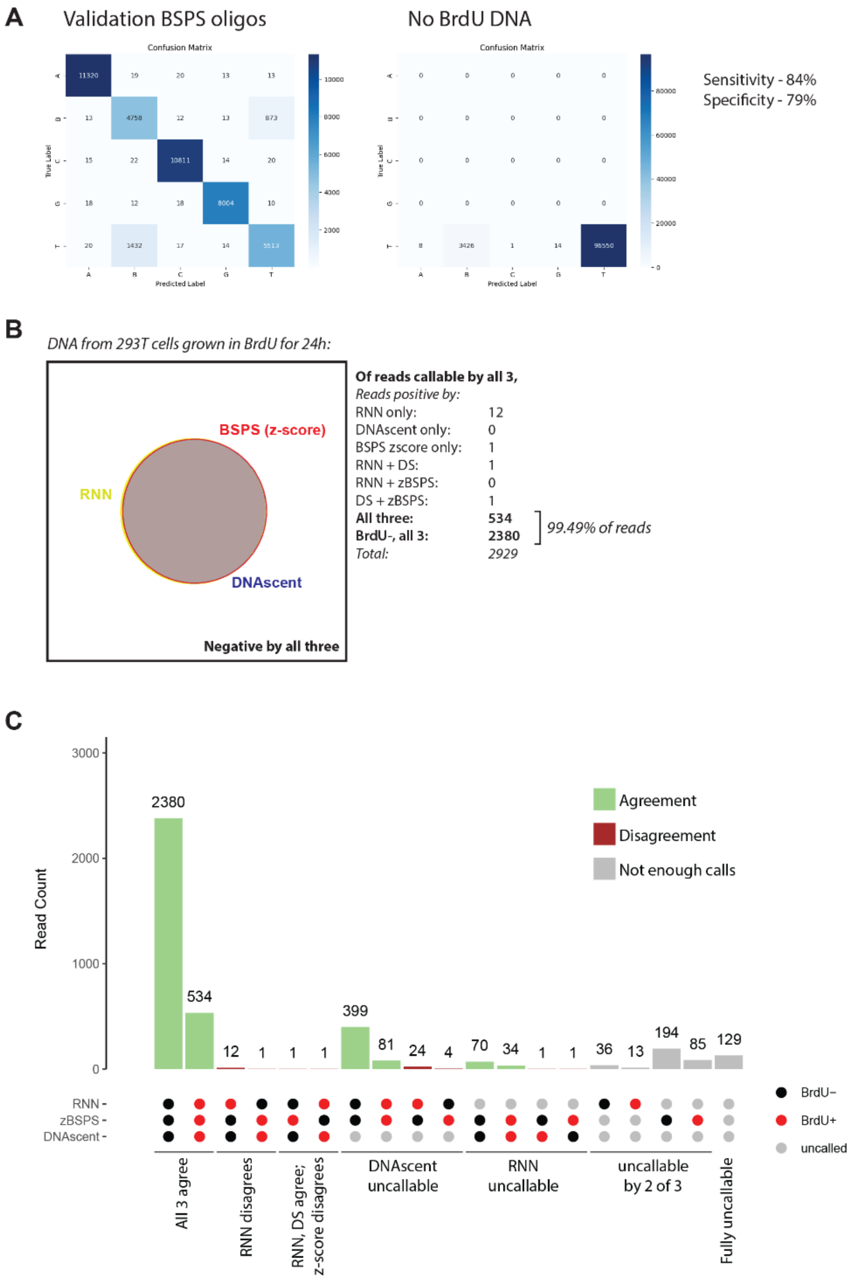

Supplementary Figure 6. Recurrent Neural Network (RNN) classification of BrdU-containing sequencing reads and comparison to other methods. (**A**) Confusion matrices display calls of RNN on validation set of BSPS oligos (left) or reads from 3T3 cells grown without BrdU (see Supplementary Fig. 4J). Calculated sensitivity and specificity and indicated. (**B**) Venn diagram depicts concordance in BrdU calling in 4,000 reads from 293T cells grown in BrdU (see Supplementary Fig. 4J) called with RNN, z-score calculated from BSPS distributions, or DNAscent v3.1.2. Only reads successfully called with all three methods are presented. The white space in the box outside the Venn diagram represents the reads called as negative by all three methods. (**C**) Upset plot depicts concordance between the three methods in the same 4,000 reads, as in (B), but now also including reads that were uncallable by one or more methods. Matrix beneath upset plot indicates BrdU status (or uncallability) from each method, and bars are colored by whether they represent agreeing calls (green), disagreeing calls (red), or calls in which only one or no methods can successfully call (gray).

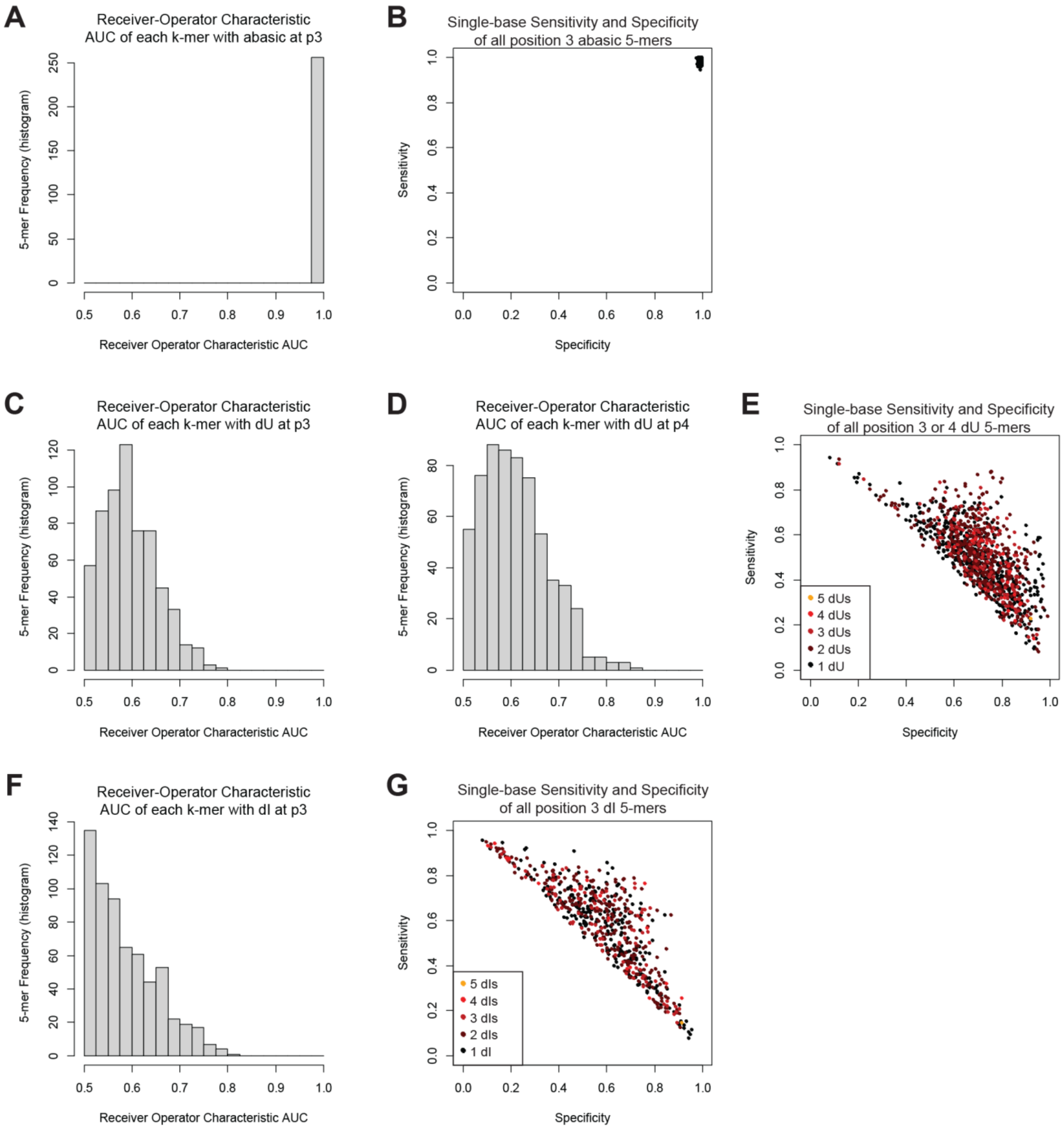

Supplementary Figure 7. Statistical analysis of single-base detection of additional modified bases. (**A**) Histogram depicts the Area Under the Curve (AUC) of the Receiver-Operator Characteristic (ROC) for the single-base detection of abasic sites in each 5-mer with an abasic site in position 3. (**B**) Scatterplot depicts the best sensitivity and specificity for each 5-mer with an abasic site in position 3, as in (B). (**C**) Histogram depicts the ROC AUC for the single-base detection of dU in each 5-mer with a dU in position 3. (**D**) Histogram depicts the ROC AUC for the single-base detection of dU in each 5-mer with a dU in position 4. (**E**) Scatterplot depicts the best sensitivity and specificity for each 5-mer with an dU site in position 3 or position 4, as in (C) and (D). (**F**) Histogram depicts the ROC AUC for the single-base detection of dI in each 5-mer with a dI in position 3. (**G**) Scatterplot depicts the best sensitivity and specificity for each 5-mer with an dI in position 3, as in (F).

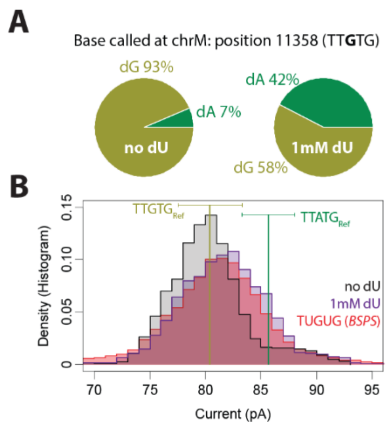

Supplementary Figure 8. dU incorporation results in predictable sequencing errors in the standard basecaller. (**A**) Pie charts depict the base call at position chrM:11358 in the sequencing reads depicted in figures 2F-J. In the dU+ condition, there is a persistent G > A miscall. (**B**) The dynamics of the current values of the fivermer at this position explain the miscalls. Position 3 has the strongest single-base effect on current. dU, unlike BrdU, results in a strong current increase even in positions 2 or 4; thus incorporation of dU here (TUGUG) results in a current increase closer to the reference sequence with dA in position 3 (TTATG) compared to dG (TTGTG), resulting in miscalling of this base.

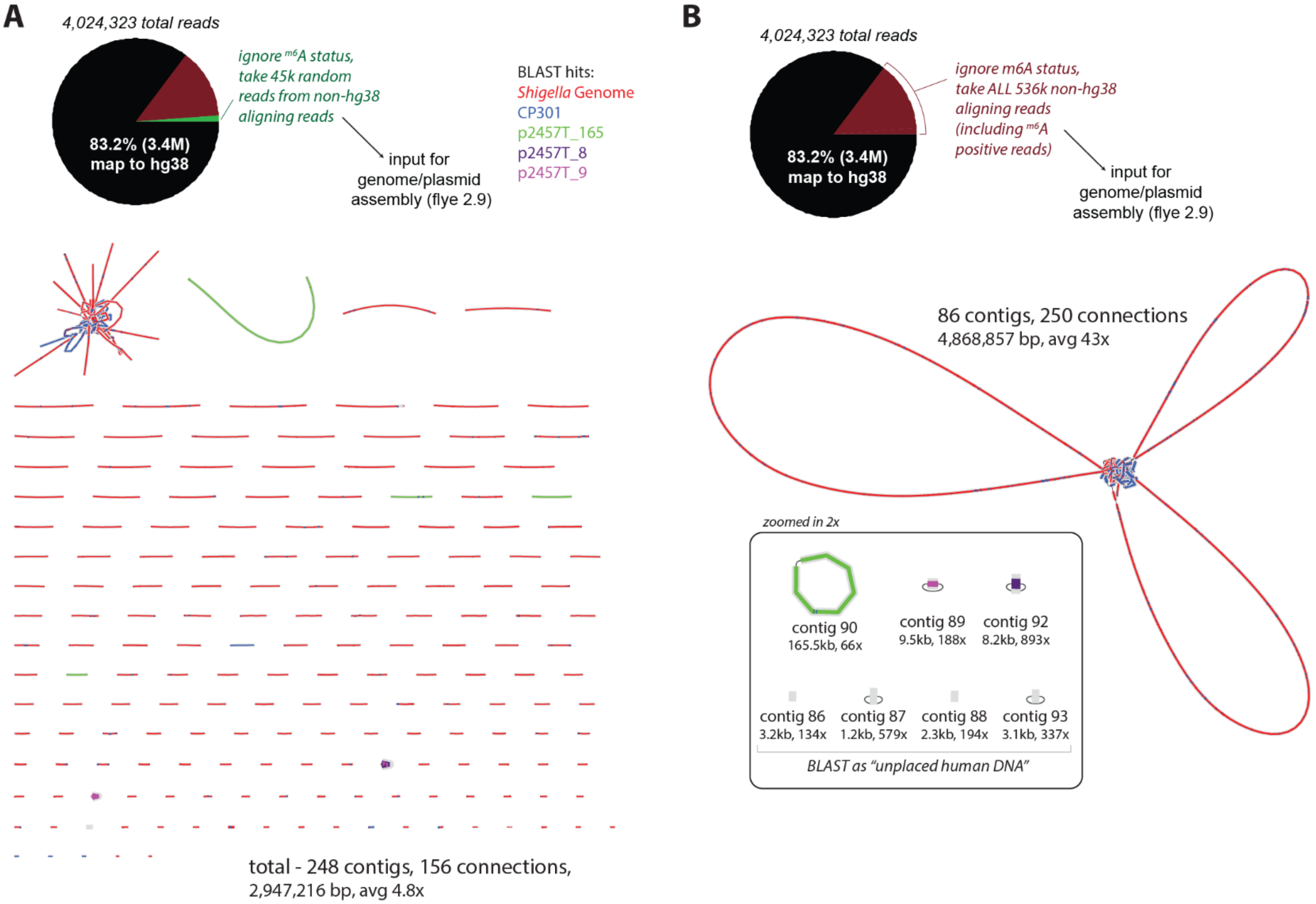

Supplementary Figure 9. Assembled *Shigella* genomes created without using ^{6m}A detection information. (**A**) In figure 4G/H, about 45,000 ^{6m}A-positive reads were used to assemble the bacterial genome. As a control, we also attempted to assemble a genome using 45,000 reads randomly selected from the non-hg38 reads. This resulted in 248 contigs, mostly corresponding to small sections of the bacterial genome. (**B**) As a second control, we also tried to assemble a genome using the entire set of non-hg38 reads (which includes the ~45,000 ^{6m}A-positive reads used in figure 4). This assembly resulted in 93 contigs; 86 of which were assembled into a large structure that comprises both the genomic chromosome and the large virulence plasmid. Three of the other contigs corresponded to known bacterial plasmids, while four new contigs appeared that, when run through BLAST against the 'nt' database, corresponded to "unplaced human DNA". Assemblies were visualized with bandage v0.8.1.

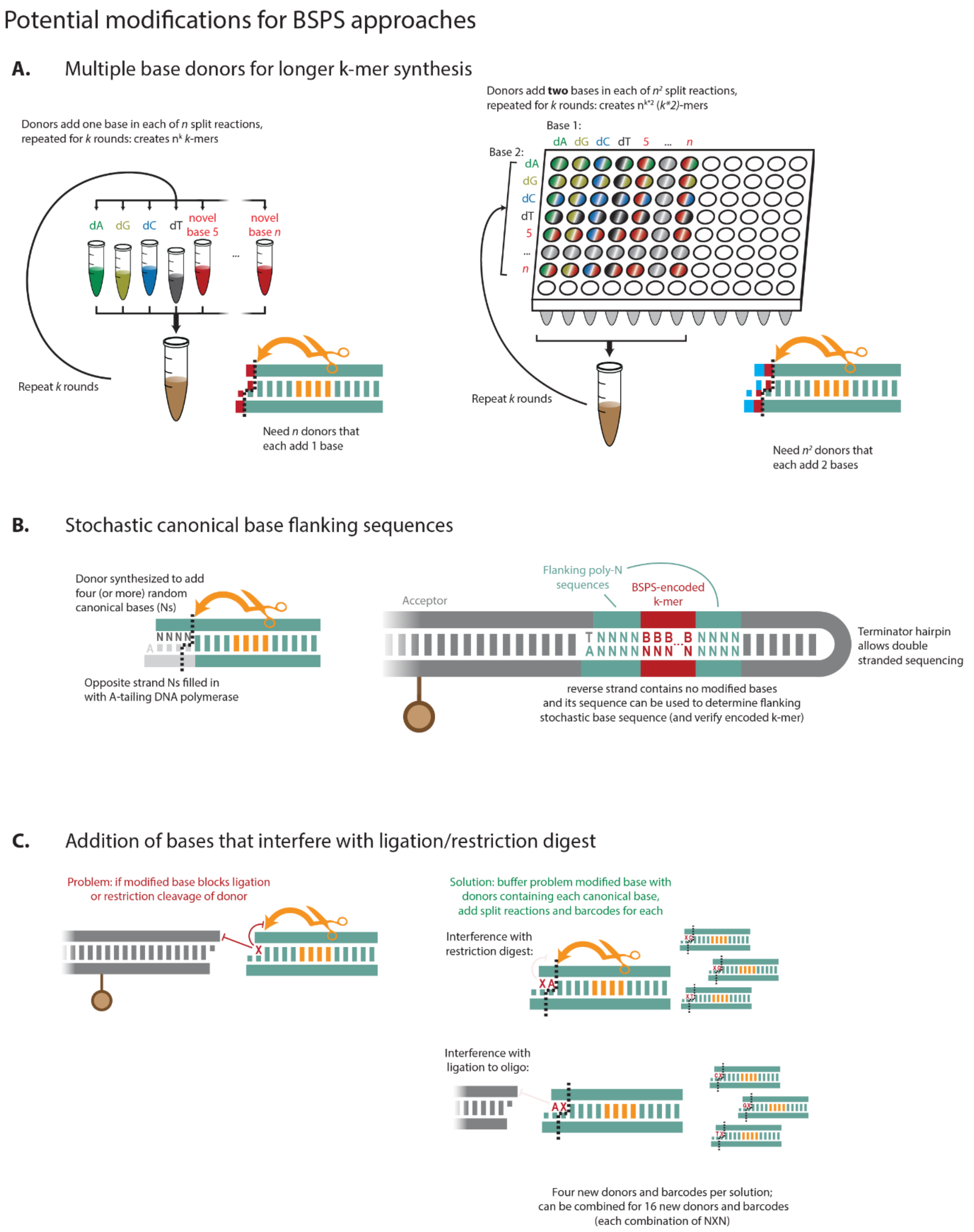

Supplementary Figure 10. Potential expansions to the BSPS approach. (**A**) In order to generate n^k *k*-mers, BSPS could be performed using *n* split reactions for *k* rounds; each split reaction would involve one base and each base would require one donor. Another potential approach would involve donors that added two bases at a time; performing n^2 split reactions for *k* rounds would create n^{2*k} (2**k*)-mers (e.g. 25 split reactions to characterize A,C,T,G and a single novel new base, for five rounds would generate all five-base 10-mers). This approach requires n^2 donors, including multiple donors for each modified base (9 donors for a single novel base, including [A,C,G,T]-base, base-[A,C,G,T] and base-base. One unique barcode would also be required for each split reaction. (**B**) In order to generate variability surrounding an encoded *k*-mer, donors can be created to add multiple random canonical bases (A,C,G,T). These would be designed so that the variability

region in the annealed oligonucleotides would be single-stranded, and filled in with the reverse complement bases by a DNA polymerase. The addition of a hairpin to the completed BSPS oligo would allow for the determination of the incorporated random bases by sequencing the reverse complement on the opposite strand, which will contain no modified bases and should be resolvable by standard basecallers. (**C**) In order to generate a BSPS library including bases that inhibit either the ligation or the restriction cleavage of the donors, buffer bases can be added on either side of the modified base, permitting the interaction and library synthesis to proceed; each new donor would require a corresponding barcode to resolve the multiple added bases.

Component	Cost
Oligos (startup)<br>*(dtBiotin acceptorR)*<br>Oligos, at scale	$900<br>*$200*<br>**$9+ base specific donor**
Consumables<br>(enzymes, plastics, etc)	$300
Sequencing reagents	$350 – 3 seq kit rxns<br>$900 – R9.4 flow cell
TOTAL	$2,150 stand-alone<br>$1,559 at scale

Supplementary Table 1. Cost estimates for a single BSPS synthesis reaction. Table presents approximate costs of reagents for a single round of BSPS synthesis, as presented in Figure 2c. Costs are based on manufacturer's list prices and exclude institutional or academic/non-profit discounts.

Modified Base	Strategy	Read count	Coverage/ kmer count	notes
**BrdU**	5 rounds, 5 bases per round<br>bc1 - dA, bc2 - dC, bc3 - dG, bc4 - dT<br>bc5 - BrdU	3,318,015<br>730,820 fully synthesized<br>(22.0%)	234X of<br>3,125 5-mers	
**dI**	5 rounds, 5 bases per round<br>bc1 - dA, bc2 - dC, bc3 - dG, bc4 - dT<br>bc5 - dI	4,976,000<br>587721 fully synthesized<br>(11.8%)	188X of<br>3,125 5-mers	
**dU**	5 rounds, 5 bases per round<br>bc1 - dA, bc2 - dC, bc3 - dG, bc4 - dT<br>bc5 - dU	4,179,952<br>664,557 fully synthesized<br>(15.9%)	213X of<br>3,125 5-mers	
**abasic site**	5 rounds; 4 rounds of 4 bases, 1 round of dU<br>bc1 - dA, bc2 - dC, bc3 - dG, bc4 - dT<br>bc5 - dU -> Abasic after UDG treatment	4,899,120<br>920,205 fully synthesized<br>(18.7%)	3,594X of<br>256 4-mers<br>(NN-abasic-NN)	high coverage variability based on k-mer sequence
**5mC(pG), 5hmC(pG)**	4 rounds; 3 rounds of 3 donors (CG, 5mCG, 5hmCG), one round of 4 donors (A,C,G,T)<br>bc1 - dA, bc2 - dC, bc3 - dG, bc4 - dT<br>bc5 - CG, bc6 - ^{5m}CG, bc7 - ^{5hm}CG	1,208,504<br>203,603 fully synthesized<br>(16.8%)	1,885X of<br>108 7-mers<br>(^{x}CGN^{x}CG^{x}CG)	synthesized as 7-mers but not analyzed as 7-mers

Supplementary Table 2. Details for generated BSPS sequencing libraries. Table presents synthesis strategy (number of bases in the k-mer and split-pool rounds performed) for each synthesized library, as well as sequencing depth and calculated frequency of correctly fully-synthesized reads (containing all barcodes and the full encoded k-mer), as well as coverage over the expected number of k-mers per experiment. Non-canonical or modified bases are presented in red.

Supplementary Note 1: Example BSPS protocol. Below is an example protocol for the synthesis of a BSPS oligo pool of an arbitrary length (number of rounds) and width (number of bases). The initial and terminal ligations are different, while intermediate ligations are similar with the exception of alternating barcode overhangs.

A. OLIGO PREPARATION

1.) Set up annealing reactions (some oligos, esp. donors, will require more than one reaction):
   a. **First round donors**:
      i. Standard Bases (A,C,T,G): donor_[base]_F + donor_[base]_r1_R)
      ii. Novel Donor[s] F&R : novel donor F + appropriate r1 R (e.g. BrdU_F + T_r1_R)
   b. **Subsequent round donors** – will need at least two annealing reactions of each:
      i. Standard Bases (A,C,T,G): donor_[base]_F + donor_[base]_n_R)
      ii. Novel Donor[s] F&R : novel donor F + appropriate n R (e.g. BrdU_F + T_n_R)
   c. **Barcodes**:
      i. Barcodes odd: bc[n]_odd_F + bc[n]_odd_R
      ii. Barcodes even: bc[n]_even_F + bc[n]_even_R
   d. **Acceptor**:
      i. Acceptor: acceptor_F + acceptor_dtBio_R
   e. **Terminator hairpins**:
      i. 5' terminator – either even or odd (depending on number of rounds): 5term_hairpin_even/odd by itself (these do not have F/R oligos)
      ii. 3' terminator – 3term_hairpin by itself

2.) Reaction mix for annealing:
   a. 1ul Annealing Buffer (2M LiCl, 0.1M Tris-HCl)
   b. 2.25 ul of each oligo (assuming 100uM stocks)
   c. Anneal in the thermocycler
      i. Non-hairpin oligos: 95°C for 5 minutes, then cool to 25°C at 1°C/min
      ii. Terminator hairpins: 95°C for 5 minutes, then place on ice until cool

3.) Phosphorylate oligos:
   a. Set up 10ul reaction, (for a 5-round synthesis – will need 3 reactions each for n donors, 1 each for others, adjust as necessary for more rounds):
      i. 2.5 ul of above annealing reaction
      ii. 1 ul 10X T4 ligation buffer (*NEB*)
      iii. 0.5 ul T4 PNK (*NEB M0201*)
      iv. 6 ul water
   b. Incubate reactions at 37°C for 30 minutes.

B. INITIAL LIGATIONS

4.) First round ligation, includes acceptor
   a. Set up 12ul reaction, one per base/barcode ("split")
      i. 4 ul annealed/phosphorylated donor oligo, unique per pool
      ii. 1 ul annealed/phosphorylated barcode **ODD** oligos, unique corresponding per base donor
      iii. 1 ul annealed/phosphorylated acceptor oligo
      iv. 6 ul Blunt-TA Ligase Master Mix (*NEB M0367*)
   b. Ligate for 60 min at 16°C, then 15 minutes at room temperature while rotating.
   c. Increase the volume to 20ul by adding 8ul water.

5.) Streptavidin Bead Binding
   a. Put one aliquot of 30 ul streptavidin beads (*Pierce 88816*) in a 1.5ml tube for each split reaction.
   b. Magnet clear and wash with 80 ul of (1X) Cutsmart (NEB) buffer.
   c. Add the 20ul ligation reactions to the beads, and add 20ul of 2X NTB buffer.
      i. 2X NTB buffer is 10mM Tris-HCl ph 8.0 + 2M NaCl
   d. Incubate while rotating for 15 min, then spin briefly.

6.) Streptavidin Bead Wash
    a. Place splits on magnet, clear, and remove and discard supernatant.
    b. Wash beads with 80 ul of 1X NTB buffer, incubate 5 minutes.
    c. Place on magnet, clear, and remove and discard supernatant.
    d. Repeat NTB wash (b + c).
    e. Wash with 40ul 1X Cutsmart buffer, clear on magnet and remove and discard supernatant.
    f. Briefly spin and remove excess supernatant.
    g. Resuspend in 50ul 1X Cutsmart
7.) Restriction enzyme cleavage of donor
    a. Add 0.5 ul of BciVI (*NEB R0596*) to each split reaction.
    b. Incubate for 15 minutes at 37°C.
    c. Clear beads on magnet, remove supernatant.
    d. Wash beads twice with 80 ul 1X NTB with five minute incubations (see 6b/c).
    e. Resuspend beads in 20.5 ul 1X T4 ligase buffer (*NEB B0202*)
        i. Note: This volume assumes the next synthesis round will have the same number of split reactions; if there will be more or less reactions, adjust this volume accordingly.

[REPEAT THE STEPS C/D FOR EACH ROUND IN THE SPLIT-POOL SYNTHESIS]

C. POOL – SPLIT

8.) Pool all split reactions in a single tube and mix thoroughly.
9.) Separate pooled mix into new separate labeled tubes (20 ul in each tube), clear on magnet and remove and discard supernatant.

D. **DONOR AND BARCODE LIGATION**

10.) Set up the (n)th ligation.
    a. Set up 10ul reaction, one per base/barcode ("split")
        i. 4 ul annealed/phosphorylated donor oligo, unique per pool
        ii. 1 ul annealed/phosphorylated barcode (**EVEN** or **ODD**, opposite of the last ligation round)
        iii. 5 ul Blunt-TA Ligase Master Mix (*NEB M0367*)
    b. Ligate for 60 minutes at 16°C then 15 minutes at room temperature while rotating.
11.) Bead Wash
    a. Place each ligation reaction on magnet, clear, and remove and discard supernatant.
    b. Wash beads with 80 ul of 1X NTB buffer, incubate 5 minutes.
    c. Place on magnet, clear, and remove and discard supernatant.
    d. Repeat NTB wash (b + c).
    e. Wash with 40ul 1X Cutsmart buffer, clear on magnet and remove and discard supernatant.
    f. Briefly spin and remove excess supernatant.
    g. Resuspend in 50ul 1X Cutsmart
    h. If this is the **LAST SYNTHESIS ROUND**, skip to section 14. Otherwise, continue to 12.
12.) Restriction enzyme cleavage of donor (**For rounds prior to the last ligation round**)
    a. Add 0.5 ul of BciVI (*NEB R0596*) to each split reaction.
    b. Incubate for 15 minutes at 37°C.
    c. Clear beads on magnet, remove supernatant.
    d. Wash beads twice with 80 ul 1X NTB with five minute incubations (see 11b/c).
    e. Resuspend beads in 20.5 ul 1X T4 ligase buffer (*NEB B0202*)
        i. Note: This volume assumes the next synthesis round will have the same number of split reactions; if there will be more or less reactions, adjust this volume accordingly.
13.) Return to Part C to pool-split for the next ligation round.

[END OF INTERMEDIATE ROUND SYNTHESIS LOOP]

E. FINAL SYNTHESIS AND CLEANUP

14.) Terminal restriction enzyme cleavage
   a. Add **0.5 ul SapI** (*NEB R0569*) to each split reaction. (Note different enzyme, this reveals the ligation site for the 3' terminator rather than for the next donor as in previous rounds)
   b. Incubate for 15 minutes at 37°C.
   c. Clear beads on magnet, remove supernatant.
   d. Wash beads twice with 80 ul 1X NTB with five minute incubations (see 11b/c).
   e. Resuspend beads in 20.5 ul 1X T4 ligase buffer (*NEB B0202*)

15.) Pool all terminal reactions in a single tube and mix thoroughly.

16.) Remove and discard supernatant, spin down and remove remaining supernatant.

17.) Resuspend beads in 5ul water.

18.) Set up hairpin terminator ligation:
   a. 4 ul of sample from previous step
   b. 10 ul of Blunt-TA Master Mix
   c. 3 ul annealed and phosphorylated 3' terminator hairpin
   d. 3 ul annealed and phosphorylated 5' terminator hairpin

19.) Ligate for 60 minutes at 16°C, then 15 minutes room temperature while rotating.

20.) Bead Wash
   a. Place each ligation reaction on magnet, clear, and remove and discard supernatant.
   b. Wash beads with 80 ul of 1X NTB buffer, incubate 5 minutes.
   c. Place on magnet, clear, and remove and discard supernatant.
   d. Repeat NTB wash (b + c).
   e. Wash with 40ul 1X Cutsmart buffer, clear on magnet and remove and discard supernatant.
   f. Briefly spin and remove excess supernatant.
   g. Resuspend in 10 ul EB (10 mM Tris-HCl, ph 8-8.5)

21.) Biotin Wash
   a. Add 20 ul 50 mM biotin solution to beads and resuspend by pipetting.
   b. Incubate at 37°C for 10 min.
   c. Add another 20 ul 50 mM biotin solution to beads and resuspendf by pipetting
   d. Incubate at 37°C for 10 minutes.
   e. Pellet on magnet, remove and keep supernatant - transfer to new tube.

22.) 2.5X SPRI/Ampure bead cleanup
   a. Add 50 ul of SPRI beads and 75 ul of bead buffer (20% polyethylene glycol (PEG) 8000 resuspended in 2.5 M NaCl)
   b. Rotate for 15 minutes at room temperature to bind DNA to beads.
   c. Clear on magnet and remove and discard supernatant.
   d. Wash beads with 175 ul 70% ethanol and then incubate for at least 1 minute.
   e. Aspirate ethanol and discard.
   f. Repeat 70% ethanol wash
   g. Aspirate ethanol and quickly spin tubes, return to magnet and remove residual ethanol with pipet
   h. Air dry for about 5 minutes.
   i. Reconstitute in 44 ul nuclease-free water, incubate at 37°C for 10 minutes.
   j. Clear on magnet, transfer supernatant to fresh tube.

23.) Digest with Exonuclease VIII (n.b. correctly synthesized oligos should have hairpins on each end and be protected from exonuclease digestion)
   a. Add 5 ul of 10X Cutsmart (NEB) to the 44ul from previous step.
   b. Add 1ul Exo VIII (*NEB M0545*)
   c. Incubate for 30 minutes at 37°C
   d. Add 1.1 ul 0.5M EDTA
   e. Inactivate for 30 minutes at 70°C
   f. Place tube on ice until cool.

24.) Repeat 2.5X SPRI/Ampure cleanup (step 22), resuspending in 45 ul nuclease-free water

25.) Restriction digest with AhdI (which will reveal a single A overhang on the 5' terminator)
   a. Add 5 ul 10X Cutsmart (NEB) to sample
   b. Add 0.5 ul AhdI (*NEB R0584*)
   c. Incubate for 15 minutes at 37°C

26.) Repeat 2.5X SPRI/Ampure cleanup (step 22), resuspending in 49ul nuclease-free water

27.) Quantify synthesized library via qubit/qPCR

F. **SEQUENCING**

Synthesized libraries are already A-tailed (due to AhdI cleavage) and do not need A-tailed or repair – for sequencing, skip those sections in the sequencing kit protocol. For instance, begin from the "Adapter ligation and clean-up" step of Oxford Nanopore's Ligation Sequencing Kit protocol.

Supplementary Note 2: Example BSPS data processing commands. Below are example R commands for the processing of a sequenced BSPS dataset.

BSPS sequencing data processing and reference current generation

BSPS reference sequences were created with the following R commands:

```
#nbases is the name of the bases (in the fasta reference name), and bases is
the expected base alignments (in this example, BrdU is named B but expected to
align to T)

nbases <- c("A","C","G","T","B")
bases <- c("A","C","G","T","T")

#ensure the barcode sequences are in the same order as the bases they encode

barcodes <-
c("GGAGGATCTCAGGTAGGACTAACCGCTAGATCTTGG","GGAGGAAGCTAGCACCATTGTGCCAAGGAATCTTGG"
,"GGAGGACCTATCCACTAGACGAGGTAATTGTCTTGG","GGAGGAGGCACAGAATGTGACCACAATGTGTCTTGG",
"GGAGGAGTAGGTAGTGCCATTCTTCGTGGATCTTGG")
i <- 1
b1 <- 1
b2 <- 1
b3 <- 1
b4 <- 1
b5 <- 1


sink("/path/to/reference.fa")

while(b1 < 6){
 while(b2 < 6){
	while(b3 < 6){
		while(b4 < 6){
			while(b5 < 6){

cat(">ref",nbases[b1],nbases[b2],nbases[b3],nbases[b4],nbases[b5],"\n",sep="")

cat("TGGTCTAGAG","ACCA",barcodes[b5],"AGAG",barcodes[b4],"ACCA",barcodes[b3],"A
GAG",barcodes[b2],"ACCA",barcodes[b1],"AGAG","GCTATTGTCTGCCCATGTGGCGCCCCAATTAGT
GACCGCACAAGAACAGTAAGGA",bases[b1],bases[b2],bases[b3],bases[b4],bases[b5],"TGTA
TGGATACTAGGGTACACAGTTGCCATTCCATAG","\n",sep="")

b5 <- b5+1
			}
			b5 <- 1
			b4 <- b4+1
		}
		b4 <- 1
		b3 <- b3+1
	}
	b3 <- 1
	b2 <- b2+1
 }
 b2 <- 1
 b1 <- b1+1
}
sink()
```

Sequenced reads were aligned to this reference using minimap2, with an increased penalty for gap opening/extension, secondary alignments disabled, and only mapping to the forward strand of the reference sequences. Reads that fully map to a reference (i.e. reads for which the entire reference sequence is represented from start to end in the read, with no gaps that could indicate barcode exclusion or mismatch) were selected from the aligned bam file using the expression (-e) flag in samtools view, with ‘rlen’ and ‘qlen’ values above a minimum length encompassing the entire acceptor, the length of the full number of barcodes,

and the encoded k-mer (e.g. longer than 60 + 5*40 + 5 = 265 for a five-mer oligo library). Full-length reads were processed via nanopolish eventalign, with the '--scale-events', '--samples' and '-n' flags enabled. Current values corresponding to the encoded k-mers were isolated from the full completed file using grep to find lines with the correct position (found in column 2 in the eventalign.txt file) corresponding to the encoded kmer (position 269 for five-mers with the references as created with the code above). Lines which corresponded to eventalign being unable to successfully align the events (e.g. "NNNNN" was returned as the 'model_kmer' in column 10) were removed, except in cases such as the abasic site library, in which the correctly sequenced modified base was expected to return an uncallable current value. The current reading values (presented in a comma-separated list in 'samples', column 14) were combined for each reference kmer (column 1), and the median and standard deviation of the current for each synthesized k-mer were calculated based on the total collection of that k-mer's current reading values.

Receiver-Operator Characteristic and 'callable k-mer' calculations

Receiver-Operator Characteristic (ROC) Area Under the Curve (AUC) values were calculated with the following R code:

```
#5mer_names.txt is a two-column text file containing an ordered list of each
of the modified base containing 5mers ($M) and the corresponding no-modified
base reference 5-mer ($R).
#5mer_samples.txt is a tab-separated table of the 'samples' values extracted
from the eventalign file, in columns with the fivemer as the first (header)
row.  5mer_samples.txt includes columns for non-modified base 5-mers (5^5 =
3,125 columns), while 5mer_names.txt does not include rows for non-modified
5-mers (5^5 - 4^5 = 2,101 rows).

base_names <- read.table("/path/to/5mer_names.txt", header=T)
data <- read.table("/path/to/5mer_samples.txt", fill=NA, sep="\t", header=T)

j <- 1
base_AUCs <- rep(0,2101)
base_max_dist <- rep(0,2101)
base_max_threshold <- rep(0,2101)
base_TPRatbest <- rep(0,2101)
base_FPRatbest <- rep(0,2101)

tempAUC <- 0
tempTPR <- 0
tempFPR <- 0

#j is kmer index, loop calculates best TPR and FPR
while(j <= 2101){
      ref_S <- data[[base_names$R[j]]]
      ref_M <- data[[base_names$M[j]]]
      ref_S <- subset(ref_S, !is.na(ref_S))
      ref_M <- subset(ref_M, !is.na(ref_M))

      data_FPR <- rep(0,length(ref_S))
      data_TPR <- rep(0,length(ref_S))

      o <- order(ref_S, decreasing=F)
      ref_S <- ref_S[o]
      tempdist <- 0
      tempthresh <- 0
      tempTPR <- 0
      tempFPR <- 0

      #i uses each reference measurement as calling threshold; this code
      assumes modified base will have a higher current.  For a decrease
      current, run this loop with '<=' (e.g. ref_M <= ref_S[i])
      i <- 1
      while(i <= length(ref_S)){
            data_TPR[i] <- mean(ref_M >= ref_S[i])
            data_FPR[i] <- mean(ref_S >= ref_S[i])

            #store the best threshold
```

```
                if(tempdist < data_TPR[i] - data_FPR[i]){
                        tempthresh <- ref_S[i]
                        tempTPR <- data_TPR[i]
                        tempFPR <- data_FPR[i]
                        tempdist <- data_TPR[i] - data_FPR[i]
                }
                i <- i+1
        }

        base_max_dist[j] <- tempdist
        base_max_threshold[j] <- tempthresh
        base_TPRatbest[j] <- tempTPR
        base_FPRatbest[j] <- tempFPR

#cycle through TPRs and FPRs to calculate AUC by trapezoid method
        tempAUC <- 0
        i <- length(ref_S)
        while(i > 1){
                tempAUC <- tempAUC + (((data_TPR[i] + data_TPR[i-1])/2 *
(data_FPR[i-1] - data_FPR[i])))
                i <- i-1
        }
        dU_AUCs[j] <- tempAUC


        j <- j+1
}
results <- data.frame(names=base_names$M, AUC=base_AUCs, max_dist =
base_max_dist, threshold = base_max_threshold, best_TPR = base_TPRatbest,
best_FPR = base_FPRatbest)
```

Monte Carlo simulations for base accuracy

Monto Carlo simulations were performed using the following R code:

```
#callableK is a unheaded text list of callable canonical base kmers ($V1) and
the threshold ($V2), as determined based on ROC AUC above.
#5mer_samples.txt is same file as above
callableK <- read.table("/path/to/base_callable_kmers.txt")
data <- read.table("/path/to/5mer_samples.txt", fill=NA, sep="\t", header=T)

MODresults <- rep(0,10000) #10,000 is the number of reads to sample
REFresults <- rep(0,10000)
i <- 1  #read index
j <- 0  #will be the randomly selected kmer from callable k-mers
n <- 15 #number of potentially modified bases per read
h <- 1  #base per read index
f <- 0.25 #replacement fraction (fraction of n’s that are modified)

while(i < 10000){
        tempref <- 0
        tempmod <- 0
        h <- 1
        while(h <= n){
                j <- sample(1:length(callableK),1)
#the following will replace each modifiable base (in this code, T) with its
modification (B) with probability f, defined above.  Change ‘T’ and ‘B’ to
the appropriate bases based on data structure.
                roll_F <- sample(1:100,1)/100
                if(roll_F <= f){
                        tempmod <- tempmod +
sample(data[[gsub('T','B',callableK$V1[j])]][!is.na(data[[gsub('T','B',callab
leK$V1[j])]])],1) - callableK$V2[j]
                }
                if(roll_F > f){
                        tempmod <- tempmod +
sample(data[[callableK$V1[j]]][!is.na(data[[callableK$V1[j]]])],1) -
callableK$V2[j]

                }
```

```
#this is the cumulative score (distance from threshold) per read -
modification is assumed to have higher current, can be changed
            tempref <- tempref +
sample(data[[callableK$V1[j]]][!is.na(data[[callableK$V1[j]]])],1) -
callableK$V2[j]

            h <- h+1
      }
      MODresults[i] <- tempmod
      REFresults[i] <- tempref
      i <- i+1
}

#calculate FPR/TPR/AUC from above simulations

i <- 0
o <- order(REFresults)
tempAUC <- 0
data_TPR <- rep(0,1001)
data_FPR <- rep(0,1001)

#calculate ROC
while(i < 1000){
      data_FPR[i] <- mean(REFresults > REFresults[o[i*10]])
      data_TPR[i] <- mean(MODresults > REFresults[o[i*10]])
      i <- i+1
}

i <- 1001
tempAUC <- 0

#calculate AUC via trapezoid method
while(i > 1){
      tempAUC <- tempAUC + (((data_TPR[i] + data_TPR[i-1])/2 * (data_FPR[i-1]
- data_FPR[i])))
      i <- i-1
}

finalAUC <- tempAUC
```

Modified base calculation from experimental data

Sequenced reads were aligned to the correct reference genome (hg38, mm10, or bacterial-specific as described in each relevant experimental section) and raw current values were generated through nanopolish eventalign, with the '-n' and '--scale-events' flags. For files expected to be very large, we piped the output to a 'cut' command to only write relevant columns (*nanopolish eventalign [flags] | cut -f 4,7,10,11,12 > thin.eventalign.txt* would be the minimum using the below code) and/or piped to a '*grep -F -f relevant_kmers.txt*' command to save only relevant kmers (callable and control).

Prior to analysis, lists of "callable" k-mers for each modified base are generated. The following code isolates only k-mers in which the presence of a modified base results in an average current level greater than two standard deviations above the reference current mean, and then calculates the fraction of these k-mers in the read that are greater than two standard deviations above the reference current; it can easily be modified to utilize the expected thresholds for each k-mer as calculated above. Also generated is a list of "control" k-mers, which are k-mers with observed currents in the BSPS library sequencing that are highly concordant with the reference current. These often don't contain a modifiable base – e.g. for a BrdU experiment, most/all of these k-mers would not contain a 'T'. The expectation is a read with significant current variance at these "control" k-mers is simply a noisy read and the variance at the control k-mers is subtracted from the variance at callable k-mers to generate the read "score".

Eventalign files were analyzed to generate read scores using the following code:

```
#for files too large for input, can also use the colClasses parameter to only
read certain columns, or process certain lines at a time

data <- read.table("/path/to/experiment_eventalign.txt", header=T)

#callable and control k-mer lists, as described above
BrdUList <- read.table("/path/to/callable_kmers_list.txt", header=F)
CtrlList <- read.table("/path/to/control_kmers_list.txt", header=F)

dataB <- subset(data, model_kmer %in% BrdUList$V1)
dataC <- subset(data, model_kmer %in% CtrlList$V1)

Readlist <- unique(dataB$read_name)
Readlist <- subset(Readlist, Readlist %in% dataC$read_name)
dataB <- subset(dataB, read_name %in% Readlist)
dataC <- subset(dataC, read_name %in% Readlist)


#the following code calculates the fraction of control and callable k-mers
that are 2x std. deviations above the expected reference mean; can also
change to calculate above threshold determined during AUC calculations
dataB$kmer_Z <- dataB$event_level_mean > (dataB$model_mean +
2*dataB$model_stdv)
readscoreB <- sapply(split(dataB$kmer_Z, dataB$read_name),mean)
dataC$kmer_Z <- dataC$event_level_mean > (dataC$model_mean +
2*dataC$model_stdv)
readscoreC <- sapply(split(dataC$kmer_Z, dataC$read_name),mean)

readscore <- readscoreB - readscoreC
```

Read scores (‘readscore’) are visualized via histogram and thresholds to determine “positive” and “negative” reads are determined based on the distribution and separation of a similarly sequenced and processed negative control sample (e.g. cells not grown in BrdU then sequenced, processed, and read scores generated for BrdU).
