# Supplementary material for: Multidimensional third-generation sequencing of modified DNA bases allows interrogation of complex biological systems
Source: Nat Commun. 2025 Jul 1;16:5676. doi: 10.1038/s41467-025-60896-x (PMC12215381; doi:10.1038/s41467-025-60896-x)
Supplement: Supplementary file 2 — Description of Additional Supplementary Information [file 41467_2025_60896_MOESM2_ESM.pdf]

Description of Additional Supplementary Files

File Name: Supplementary Data 1
Description: Table presents sequences of oligos used in BSPS synthesis protocol; including acceptors, base donors, barcodes (even and odd), and 3’ and 5’ terminator hairpins.

File Name: Supplementary Data 2
Description: Table presents BSPS-derived current values for all deoxyinosine containing fivemers, sequenced on an R9.4.1 flow cell. Columns are as follows: fivemer (encoded fivemer), I (observed current, pA), sd (standard deviation of observed current), evt_count (event count; total number of current measurements taken of each fivemer), read_count (number of reads covering each fivemer), noI (fivemer with dIs replaced with dG), noIcurrent (current of noI fivemer).

File Name: Supplementary Data 3
Description: Table presents BSPS-derived current values for all bromodeoxyuracil (BrdU) containing fivemers, sequenced on an R9.4.1 flow cell. Columns are as follows: fivemer (encoded fivemer), I (observed current, pA), sd (standard deviation of observed current), evt_count (event count; total number of current measurements taken of each fivemer), read_count (number of reads covering each fivemer), noB (fivemer with BrdUs replaced with dT), noBcurrent (current of noB fivemer).

File Name: Supplementary Data 4
Description: Table presents BSPS-derived current values for all deoxyuracil containing fivemers, sequenced on an R9.4.1 flow cell. Columns are as follows: fivemer (encoded fivemer), I (observed current, pA), sd (standard deviation of observed current), evt_count (event count; total number of current measurements taken of each fivemer), read_count (number of reads covering each fivemer), noU (fivemer with dUs replaced with dT), noUcurrent (current of noU fivemer).

File Name: Supplementary Data 5
Description: Table presents BSPS-derived current values for all abasic site containing fivemers, sequenced on an R9.4.1 flow cell. Columns are as follows: fivemer (encoded fivemer), I (observed current, pA), sd (standard deviation of observed current), evt_count (event count; total number of current measurements taken of each fivemer), read_count (number of reads covering each fivemer).
