# Supplementary material for: Multidimensional third-generation sequencing of modified DNA bases allows interrogation of complex biological systems
Source: Nat Commun. 2025 Jul 1;16:5676. doi: 10.1038/s41467-025-60896-x (PMC12215381; doi:10.1038/s41467-025-60896-x)
Supplement: Supplementary file 8 — Reporting Summary [file 41467_2025_60896_MOESM8_ESM.pdf]

nature portfolio

Corresponding author(s): William Flavahan

Last updated by author(s): 6/05/2025

Reporting Summary

Nature Portfolio wishes to improve the reproducibility of the work that we publish. This form provides structure for consistency and transparency in reporting. For further information on Nature Portfolio policies, see our Editorial Policies and the Editorial Policy Checklist.

Statistics

For all statistical analyses, confirm that the following items are present in the figure legend, table legend, main text, or Methods section.

n/a	Confirmed	
☐	☒	The exact sample size (n) for each experimental group/condition, given as a discrete number and unit of measurement
☐	☒	A statement on whether measurements were taken from distinct samples or whether the same sample was measured repeatedly
☐	☒	The statistical test(s) used AND whether they are one- or two-sided<br>*Only common tests should be described solely by name; describe more complex techniques in the Methods section.*
☐	☒	A description of all covariates tested
☐	☒	A description of any assumptions or corrections, such as tests of normality and adjustment for multiple comparisons
☐	☒	A full description of the statistical parameters including central tendency (e.g. means) or other basic estimates (e.g. regression coefficient) AND variation (e.g. standard deviation) or associated estimates of uncertainty (e.g. confidence intervals)
☐	☒	For null hypothesis testing, the test statistic (e.g. F, t, r) with confidence intervals, effect sizes, degrees of freedom and P value noted<br>*Give P values as exact values whenever suitable.*
☒	☐	For Bayesian analysis, information on the choice of priors and Markov chain Monte Carlo settings
☒	☐	For hierarchical and complex designs, identification of the appropriate level for tests and full reporting of outcomes
☒	☐	Estimates of effect sizes (e.g. Cohen's d, Pearson's r), indicating how they were calculated

Our web collection on statistics for biologists contains articles on many of the points above.

Software and code

Policy information about availability of computer code

Data collection	Sequencing data was collected on a Nanopore minION mk1C using the then-current version of MinKNOW as provided by the manufacturer.
Data analysis	As described in the methods section of the manuscript, data was analyzed using the following programs. Basecalling: Guppy (v6.0.7) and Dorado (v0.6.0); Fast5 file processing: ont_fast5_api (v3.1); Nanopore methylation calls: modkit (v 0.2.4), Nanopore current extraction: Nanopolish (v0.14.0); Alignment: minimap2 (v2.24) and Samtools (v1.16); bacterial genome assembly: Flye (v 2.9.3), visualization with bandage (v0.8.1). Data were visualized and statistical calculations performed in R (v4.1.1). DNAscent (v3.1.2) was used for the comparative BrdU calling analysis in the extended data. The code used for recurrent neural network analysis of BrdU is available on GitHub: https://github.com/haibol2016/NanoporeBrdUCaller or https://doi.org/10.5281/zenodo.15593740. Example R commands to process and analyze a sequenced BSPS library, or to perform statistical analysis of BSPS data, is included with the manuscript as supplementary notes 1 and 2.

For manuscripts utilizing custom algorithms or software that are central to the research but not yet described in published literature, software must be made available to editors and reviewers. We strongly encourage code deposition in a community repository (e.g. GitHub). See the Nature Portfolio guidelines for submitting code & software for further information.

Data

Policy information about availability of data

All manuscripts must include a data availability statement. This statement should provide the following information, where applicable:

- Accession codes, unique identifiers, or web links for publicly available datasets
- A description of any restrictions on data availability
- For clinical datasets or third party data, please ensure that the statement adheres to our policy

The data and materials availability statement in the manuscript is as follows: All processed data are available in the main text or the supplementary materials and data, or the source data file. The raw sequencing reads (Oxford Nanopore fast5 files) generated in this study have been deposited into Sequencing Read Archive (SRA) under accession code PRJNA1083468, https://www.ncbi.nlm.nih.gov/bioproject/PRJNA1083468. An example protocol and example R commands to generate and process a sequenced BSPS library are included as supplementary notes 1 and 2. Source data are provided with this paper.

Research involving human participants, their data, or biological material

Policy information about studies with human participants or human data. See also policy information about sex, gender (identity/presentation), and sexual orientation and race, ethnicity and racism.

Reporting on sex and gender	n/a
Reporting on race, ethnicity, or other socially relevant groupings	n/a
Population characteristics	n/a
Recruitment	n/a
Ethics oversight	n/a

Note that full information on the approval of the study protocol must also be provided in the manuscript.

Field-specific reporting

Please select the one below that is the best fit for your research. If you are not sure, read the appropriate sections before making your selection.

[x] Life sciences   [ ] Behavioural & social sciences   [ ] Ecological, evolutionary & environmental sciences

For a reference copy of the document with all sections, see nature.com/documents/nr-reporting-summary-flat.pdf

Life sciences study design

All studies must disclose on these points even when the disclosure is negative.

Sample size	No statistical methods were used to predetermine sample sizes.
Data exclusions	Reads from the sequencer that failed quality checks at the manufacturer's default settings (e.g. due to poor read quality or minimum length filters) were excluded from analysis. QC-passed reads which then failed to align to the reference genome (hg38, mm10, S. flexneri NC_004741.1, or the assembled bacterial contigs) were excluded from analysis in experiments that required genome alignment (all but the bacterial genome assembly). No other data were excluded from analyses.
Replication	All findings were consistent in all experimental replicates tested, as presented in the data with the manuscript (no replicates were excluded from analyses/figures).
Randomization	Experiments were performed in commercially-available lines derived from a single population (e.g. tissue culture passage/dish) prior to being separated/split for each experimental replicate; no human or animal specimens which could be randomized were studied.
Blinding	Investigators were not blinded to the sample origins during data processing.

Reporting for specific materials, systems and methods

We require information from authors about some types of materials, experimental systems and methods used in many studies. Here, indicate whether each material, system or method listed is relevant to your study. If you are not sure if a list item applies to your research, read the appropriate section before selecting a response.

Materials & experimental systems

n/a	Involved in the study
☒	☐ Antibodies
☐	☒ Eukaryotic cell lines
☒	☐ Palaeontology and archaeology
☒	☐ Animals and other organisms
☒	☐ Clinical data
☒	☐ Dual use research of concern
☒	☐ Plants

Methods

n/a	Involved in the study
☒	☐ ChIP-seq
☒	☐ Flow cytometry
☒	☐ MRI-based neuroimaging

Eukaryotic cell lines

Policy information about cell lines and Sex and Gender in Research

Cell line source(s)	As described in the methods and materials section of the manuscript, the following cell lines from the following sources/identifiers were used: LN229 (ATCC CRL-2611), T98G (ATCC CRL-1690), BT142 (ATCC, ACS-1018), T84 (ATCC CCL-248), 293T (ATCC, CRL-3216) and 3T3 (ATCC CCL-92). Mitochondrial "Cybrid" cells (143B cells with either WT or KSS-associated mitochondrial mutations) were provided by Dr. Brett Kaufman at University of Pittsburgh. The following bacterial strain was utilized in this study: Shigella flexneri type 2A strain 2457T (ATCC, 700930).
Authentication	Cell lines were authenticated via STR profiling.
Mycoplasma contamination	Cell lines were confirmed to be mycoplasma-free by PCR testing performed at least every three months while the lines were in culture. Additionally, all sequencing reads from each experiment/sequencing run were aligned against the genomes of the eight most common laboratory-related mycoplasma strains to confirm the lack of mycoplasma-derived DNA in our sequenced datasets.
Commonly misidentified lines (See ICLAC register)	No commonly misidentified lines were used in this study.

Plants

Seed stocks	n/a
Novel plant genotypes	n/a
Authentication	n/a
